# Human papillomavirus infection in Beijing, People's Republic of China: a population-based study

**DOI:** 10.1038/sj.bjc.6605351

**Published:** 2009-09-29

**Authors:** R Zhao, W Y Zhang, M H Wu, S W Zhang, J Pan, L Zhu, Y P Zhang, H Li, Y S Gu, X Z Liu

**Affiliations:** 1 Beijing Obstetric and Gynecology Hospital, Capital Medical University, No. 251 Yao Jiayuan Road, Chaoyang District, Beijing 100026, China; 2 Beijing Haidian Maternal and Child Health Care Hospital, 35 Hai Diannan Road, Haidian District, Beijing 100080, China; 3 Beijing Chaoyang Maternal and Child Health Care Hospital, 25 Hua Weili Road, Chaoyang District, Beijing 100020, China; 4 Beijing Tongzhou Maternal and Child Health Care Hospital, 38 Yu Qiaozhong Road, Tongzhou District, Beijing 101101, China

**Keywords:** human papillomavirus, cervical neoplasia, China, epidemiology

## Abstract

**Background:**

No recent data exist on human papillomavirus (HPV) infection in Beijing, People's Republic of China.

**Materials and method:**

We interviewed and examined a representative, randomly selected sample of 5552 sexually active women aged 25–54 years. Cervical cell samples were analysed for HPV DNA by a MY09/11-based PCR assay.

**Results:**

Human papillomavirus prevalence was 6.7% overall and 4.8% among women without cervical abnormalities. Of the 21 subtypes identified, HPV16 was the commonest type (2.6% overall; 39.1% of HPV-positive women), followed by HPV 58 (1.0%), 33 (0.8%), 43 (0.7%) and 56 (0.7%). High-risk HPV types predominated in all age groups. Human papillomavirus prevalence was highest in young to middle-aged women. Marital status, number of husband's sexual partners, age at sexual debut and nulligravidity were all associated with being HPV positive.

**Conclusions:**

In our survey, HPV 16, HPV 58 and HPV 33 were the most prevalent HPV types in Beijing, indicating the potential for the prophylactic HPV 16/18 vaccine in China.

Human papillomavirus (HPV) has been established as a necessary cause of cervical cancer, found in 99.7% of invasive cervical carcinomas in the best-studied series (Bosch *et al*, 2002; Woodman *et al*, 2007). Prevalence of genital HPV infections and type-specific distribution vary greatly by geographical region (Clifford *et al*, 2005; de Sanjose *et al*, 2007). Therefore, data on HPV type-specific prevalence among women in different populations are important in elucidating the impact of an HPV vaccine and HPV-based screening in those populations.

The People's Republic of China, with a population of about 1.3 billion, is the world's most populous country. Although Chinese women have been considered to have a relatively low risk (LR) of developing cervical cancer, there are approximately 100 000 new cases and 30 000 deaths per year in China (Parkin *et al*, 2002, 2005). In Beijing, the capital of China, the population grew to 15.4 million in 2005, a robust annual increase of 2.1% (National Bureau of Statistics of China Online, http://www.stats.gov.cn). However, despite representing an appreciable portion of the nation's population, no recent data on HPV prevalence and type-specific distribution exist for Beijing women.

In this study, we report a population-based study of the type-specific and age-specific distribution of cervical HPV infection and of its risk factors in Beijing, China.

## Materials and methods

We carried out the present survey between September 2006 and December 2008 in Beijing, which is the country's political, economic and cultural centre; it consists of 18 districts, four of which are urban, four suburban and 10 outer suburban. A total of 5681 women aged 25–54 years were recruited from the population list of four urban, four suburban and four randomly chosen outer suburban districts in Beijing, China. On the basis of data from the 2005 census published by the National Bureau of Statistics of China, our representative sample of the adult female population of Beijing was stratified by age and regional distribution using a four-stage cluster sampling method. In the first stage, 12 of the 18 districts of Beijing were randomly selected. In the second stage, 4–20 townships were randomly selected from each district according to the proportion of each district's population. Then in the third stage, one neighbourhood was randomly extracted from each township. Finally, from the lists provided by local neighbourhoods, we derived a final random sample of 50 women per neighbourhood. On the basis of the age ratios of the 2005 census, we strove to enroll approximately 10 women aged 25–34 years, 14 women aged 35–44 years, 13 women aged 45–54 years and 13 migrant women aged 25–54 years from each neighbourhood.

According to the inclusion criteria, women who (1) lived in Beijing for more than half a year; (2) had no history of cervical surgery; (3) were not pregnant; (4) had no history of pelvic radiation therapy; (5) did not have a gynaecological examination and/or treatment in the past 2 years; and (6) were physically and mentally able to undergo an interview and a pelvic examination were invited to participate in this study. After selecting our randomised sample of the population, we made house-to-house visits to recruit these women and invite them to visit their local Maternal and Child Health Hospitals to participate in this study. Here, the interview was administered to the women by two–four experienced gynaecologists, covering socio-demographic information and obstetric and gynaecological history, including number of sexual partners (and also of the husband); participants then had a gynaecological examination and a cervical cell sample taken for HPV DNA analysis.

Written consent was obtained from all participants according to the regulations of the Beijing O&G hospital and 12 various district Maternal and Child Health Clinics, which approved the study.

A total of 5681 women underwent a pelvic examination; a sample of exfoliated cervical cells for liquid-based cytology and HPV testing was collected later. First, a cytobrush was inserted into the endocervical canal, and rotated gently in a clockwise direction five times. A cytobrush was then inserted into the bottom of a preservation solution vial (Thinprep, CytycCorp, Boxborough, MA, USA) and swirled 10 times vigorously. Finally, the brush was discarded and the vial contents were sent to Beijing O&G Hospital for cytological analysis. All smears were reported by two cyto-technicians separately, the abnormal/indeterminate ones were reviewed by a senior cytologist for final diagnosis. The results were classified according to the Bethesda System. All women with an abnormal cytological test (ASCUS or more severe) were referred to the study colposcopist and a biopsy was performed. All abnormal smears and 10% of normal smears chosen at random were sent for review by an experienced cytopathologist. Cervical biopsies were reviewed by a senior pathologist. All confirmed or highly suspicious high-grade or invasive lesions were treated at the Beijing O&G Hospital with loop excision, surgical conisation, hysterectomy or radiotherapy, according to protocols. In this study, cervical abnormalities were defined as the presence of histologically confirmed cervical intra-epithelial neoplasia (CIN) 1 or worse.

DNA was extracted first from the remainder of the Thinprep sample. To confirm the presence of human DNA, *β*-globin PCR analysis was performed on each sample and only *β*-globin-positive samples were included in further analyses.

The overall presence of HPV DNA positivity was determined by PCR using 5′-biotinylated MY09/11 consensus primers, as described previously (Coutlee *et al*, 1999). The presence of 23 subtypes, including HPV 16, 18, 31, 33, 35, 39, 45, 51, 52, 53, 56, 58, 59, 66, 68, 73, 83, MM4, 6, 11, 42, 43 and 44, was investigated by reverse dot blot hybridisation of the PCR product, as described in previous studies (Kawasaki and Chehab, 1994; Coutlee *et al*, 1999). High-risk (HR) HPV types for this analysis included HPV types 16, 18, 31, 33, 35, 39, 45, 51, 52, 56, 58, 59, 68, 73 and 82 (Munoz *et al*, 2003). All other HPV types were considered LR. Multiple HPV infections with the presence of at least one HR type were considered as HR.

### Statistical analysis

Statistical analysis was performed using SPSS version 15 software. Odds ratios (ORs) for HPV positivity and corresponding 95% confidence intervals (CIs) were calculated using unconditional, logistic regression, adjusted for age groups (25–29, 30–34, 35–39, 40–44, 45–49 and 50–54 years). The statistical significance of trends for ORs (*P* for trend) was assessed by treating ordinal variables as continuous.

## Results

Of the 5681 women who provided cervical cell samples, 32 had inadequate cytology results and 97 had *β*-globin-negative samples, leaving 5552 women with valid cytological and HPV results. Among these, 268 (4.8%) had histologically confirmed cervical abnormalities, including 210 CIN1, 37 CIN2, 18 CIN3 and 3 microinvasive carcinoma. Meanwhile, HSIL was diagnosed in 0.27% (*n*=15) and the proportion of HSIL that was histologically confirmed was 93.3%. The mean age of the total screened population was 39.7 years.

Overall, the prevalence of HPV of any subtype was 6.7% (4.8 and 44.4% among women with normal and abnormal cervical findings, respectively, Table 1). A total of 229 women (4.1% overall, 61.7% of HPV-positive women) had single-type infection and a total of 142 women (2.6%, 38.3%) had multiple HPV infections. In all, 21 individual types were identified. High-risk HPV infections were more frequent (5.8% of all women) than LR infections (2.0%). The most commonly found types in either single or multiple infections were HPV 16 (HR) (2.6%), HPV 58 (HR) (1.0%), HPV 33 (HR) (0.8%), HPV 43 (0.7%) (an LR type, 41 women) and HPV 56 (HR) (0.7%). High-risk HPV was found in 38.8% of women with cervical abnormalities, and in only 4.1% of normal women. The proportion of HPV 16 and HPV 18, both of which are included in currently licensed prophylactic HPV vaccines, was 45.8% among HPV-positive women and their prevalence was 85.7% for pathological grades of CIN 3 or worse.

**Figure 1:**
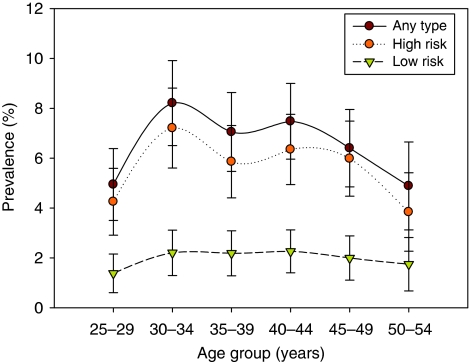
Age-specific prevalence of human papillomavirus DNA and corresponding 95% confidence interval (Beijing, China, 2008).

**Figure 2:**
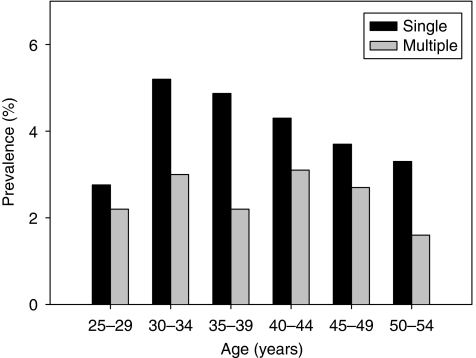
Percentage of women in each age group infected with single and multiple human papillomavirus.

Figure 1 shows the age distribution of HPV prevalence (any type, HR and LR types separately). Age-specific prevalence increased from 4.9% at age 25–29 years to a peak of 8.2% at age 30–34 years and then decreased to 7.1% at age 35–39 years. Among women aged 40–54 years, prevalence decreased progressively from 7.5% at 40–44 years to 6.4 and 4.9% at ages 45–49 and 50–54 years, respectively. Age-specific prevalence in Beijing peaked at 8.2% at 30–34 years. High-risk types were common in women aged 30–34 years, and then gradually decreased. In contrast, for LR prevalence, there was no significant age-specific trend. The proportion of HPV-positive samples infected with only one HPV type was highest at 30–34 years, whereas the proportions with multiple types at 30–34- and 40–44-year age groups were 3.0 and 3.1%, respectively, which were higher than other age groups (Figure 2).

Table 2 show the relationship between HPV positivity and some major characteristics of this population after adjustment for age. Smoking women were associated with a higher HPV prevalence than were never smokers (OR=1.25, 95% CI: 1.01–1.56). Unmarried women were more likely to be infected with HR (OR=1.63, 95% CI: 1.07–2.49) and LR HPV (OR=1.54, 95% CI: 0.77–3.09) than married women. Most women (96.9%) reported having only one sexual partner in their life; those with two or more lifetime sexual partners seemed to have an increased risk of HR HPV positivity (OR=1.45, 95% CI: 1.06–2.41). The prevalence of HPV was significantly increased with the number of husband's sexual partners (OR for two partners *vs* one=1.66, 95% CI: 1.07–2.57; OR for three partners or more *vs* one=3.67, 95% CI: 0.77–17.43). The increasing age at first intercourse was inversely associated with being HPV positive (OR for ⩽20 *vs* ⩾28 years=1.74, 95% CI: 1.14–2.65; OR for 21–27 *vs* ⩾28 years=1.10, 95% CI: 0.84–1.46).

Nulliparous women were more likely to be infected with HR HPV than women who had been pregnant earlier (OR=2.15, 95% CI: 1.16–4.00). However, there was no association of birth numbers with HPV positivity (data not shown). Age at first pregnancy was unrelated to HPV infection, whereas a trend of increased HPV positivity with younger age at first pregnancy was seen (OR for ⩽24 *vs* ⩾30 years=2.09, 95% CI: 1.01–4.32; OR for 25–29 *vs* ⩾30 years=2.00, 95% CI: 0.97–4.12). Oral contraceptive use was not associated with HR and LR HPV infections, but condom use was somewhat protective against HPV infection. No significant association was found between HPV positivity and race, occupation, education level, age at menarche, marriage or menopause, or history of spontaneous or voluntary abortion (data not show). In addition to age, when marital status, number of sexual partners, husband's sexual partners and smoking were included in the same multiple logistic regression, the corresponding ORs did not change materially.

**Figure 3:**
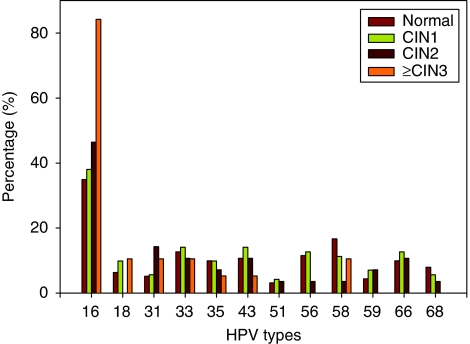
Human papillomavirus type-specific distribution and pathology grade (Beijing, China, 2008).

Figure 3 shows the percentage of specific HPV types in different pathological grades. There was an obvious increasing percentage with increasing pathology for HPV 16 and the highest percentage was in samples worse than CIN3. For HPV 18, the highest prevalence was also seen in samples worse than CIN3. For HPV types 35, 58 and 68, the highest proportion was observed in the normal group, and for HPV types 33, 43, 56 and 66, it was observed in the CIN1 group.

## Discussion

Among this representative sample of Beijing women, HPV 16 was the most frequently detected type, as found in many previous studies in different world regions (Clifford *et al*, 2005). Nevertheless, its overall prevalence was relatively low (6.7%), which correlates with the low incidence of cervical cancer in China (Yang *et al*, 2003). Meanwhile, a large proportion of women with only one sexual partner (96.9%) in this general population was probably an important cause of low HPV prevalence in Beijing. In previous Asian studies, HPV prevalence was lower, such as in Vietnam (Hanoi, 2.0%; Pham *et al*, 2003), Thailand (Lampang, 9.1%; Sukvirach *et al*, 2003) and Indonesia (11.4%; Vet *et al*, 2008), compared with countries such as Nigeria (Ibadan, 26.3%; Thomas *et al*, 2004) and Chile (29.2%; Ferreccio *et al*, 2008).

Besides HPV 16, HR HPV 58 and 33 were also common in this general population, followed by LR HPV 43 and HR HPV 56. Surveys have reported relatively high HPV 58 and HPV 33 prevalence in Asia (de Sanjose *et al*, 2007). HPV 16, 18, 58, 33, 52 and 45 were commoner in Asia than in other areas (Bao *et al*, 2008b). The predominance of HPV 58 and HPV 33 in Beijing is in accordance with the results of certain surveys in other regions of China (Dai *et al*, 2006; Li *et al*, 2006; Bao *et al*, 2008a).

In our survey, HPV prevalence was highest in women aged 30–34 years and declined thereafter. This age-specific pattern is similar to some previous studies in China (Dai *et al*, 2006), but differs somewhat from other surveys elsewhere (Munoz *et al*, 2004; Hibbitts *et al*, 2006). In China, which is a relatively conservative society, late marriage and late childbirth are encouraged. The relatively late age at first intercourse (mean: 24.0 years), along with a tendency to delay marriage and childbirth, may explain the relatively high HPV prevalence in young to middle-aged women in Beijing. Furthermore, persistent infections are reported to gradually become more prominent with age (Castle *et al*, 2005). In our survey, the prevalence of multiple HPV infection is high in women aged 30–34 years. Thus, the high HPV prevalence in young to middle-aged women in Beijing may indicate a relative lack of viral clearance or a reactivation of latent infection, which formed co-infection.

The number of husband's sexual partners was strongly associated with HPV infection, indicating the important role of males in HPV transmission to female partners in the Beijing population, among whom the majority (96.9%) have only one sexual partner in their life. As expected, marital status of women and age at first intercourse were risk factors for HPV infection. Similar to previous studies (Deacon *et al*, 2000; Munoz *et al*, 2002; Pham *et al*, 2003; Shin *et al*, 2003), we found that nulliparous women had a higher prevalence of HPV than women who had been pregnant earlier. However, there was no trend with the number of pregnancies, as found in some studies (Thomas *et al*, 2004). We found an association between smoking and HPV prevalence as in many other (Sukvirach *et al*, 2003; Vaccarella *et al*, 2008), though not all, studies (Harris *et al*, 2004). The use of oral contraceptives was not associated with HPV infection in our study, but we had no details of duration of use; at present, the question of an association is unclear (Green *et al*, 2003). Previous findings are also inconsistent for the association between condom use and HPV infection (Manhart and Koutsky, 2002; Vaccarella *et al*, 2006); our data indicated a possible limited protective trend.

Comparisons of the distribution of common HPV types among pathological grades, HPV 16 and HPV 18 were frequent with CIN3/CC relative to their prevalence in normal, CIN1 and CIN2 grades, reflecting their predominant roles in invasive carcinomas in Beijing. In most countries, HPV 16 is by far the most common type in cervical carcinoma, followed by HPV 18 (Bosch *et al*, 2008; Zhao *et al*, 2008; Cai *et al*, 2009). It would seem that the prophylactic vaccine against HPV 16/18 will also be effective in reducing the cervical cancer burden in Beijing, as the prevalence of these two types was 85.7% for pathological grades of CIN 3 or worse.

In conclusion, the commonest HPV types in the general female population of Beijing are HPV 16, 58 and 33, and the HPV 16/18 vaccination is expected to substantially reduce the cervical cancer burden in China.

## Figures and Tables

**Table 1:** Prevalence of various human papillomavirus (HPV) types by histological findings among 5552 women (Beijing, China, 2008)

	**Cervical abnormalities**								
	**Absent**			**Present^b^**			**Total**		
**HPV type^a^**	**Single**	**Multiple**	**Total (%)**	**Single**	**Multiple**	**Total (%)**	**Single**	**Multiple**	**Total (%)**
Negative			5032 (95.2)			149 (56.0)	—	—	5181 (93.3)
									
*Positive*									
Any	159	93	252 (4.8)	70	49	119 (44.4)	229	142	371 (6.7)
High risk	124	92	216 (4.1)	57	47	104 (38.8)	181	139	320 (5.8)
Low risk	35	43	78 (1.5)	13	20	33 (12.3)	48	63	111 (2.0)
									
*High risk*									
16	51	37	88 (1.7)	30	27	57 (21.3)	81	64	145 (2.6)
58	20	22	42 (0.8)	5	7	12 (4.5)	25	29	54 (1.0)
33	6	26	32 (0.6)	2	13	15 (5.6)	8	39	47 (0.8)
56	11	18	29 (0.5)	3	7	10 (3.7)	14	25	39 (0.7)
35	6	19	25 (0.5)	0	10	10 (3.7)	6	29	35 (0.6)
18	4	12	16 (0.3)	3	6	9 (3.4)	7	18	25 (0.5)
68	8	12	20 (0.4)	2	3	5 (1.9)	10	15	25 (0.5)
31	5	8	13 (0.2)	4	6	10 (3.7)	9	14	23 (0.4)
59	4	7	11 (0.2)	2	5	7 (2.6)	6	12	18 (0.3)
51	5	3	8 (0.2)	2	2	4 (1.5)	7	5	12 (0.2)
39	2	0	2 (0.03)	3	2	5 (1.9)	5	2	7 (0.1)
45	2	2	4 (0.1)	1	1	2 (0.8)	3	3	6 (0.1)
73	0	4	4 (0.1)	0	0	0	0	4	4 (0.1)
52	0	1	1 (0.02)	0	0	0	0	1	1 (0.02)
									
*Low risk*									
43	8	19	27 (0.5)	5	9	14 (5.2)	13	28	41 (0.7)
66	13	12	25 (0.5)	5	7	12 (4.5)	18	19	37 (0.7)
42	4	7	11 (0.2)	0	3	3 (1.1)	4	10	14 (0.3)
53	3	6	9 (0.2)	2	2	4 (1.5)	5	8	13 (0.2)
11	4	1	5 (0.1)	0	1	1 (0.4)	4	2	6 (0.1)
83	3	2	5 (0.1)	0	0	0	3	2	5 (0.1)
6	0	2	2 (0.03)	1	1	2 (0.8)	1	3	4 (0.1)

a The same women can be counted more than once for multiple infection.

b Includes all histologically confirmed CIN1 and worse.

**Table 2:** Detection of cervical human papillomavirus (HPV) DNA according to major risk factors among 5552 women (Beijing, China, 2008)

		**HPV DNA positive**	
	**Total no.**	**Number (%)**	**OR^a^ (95% CI)**
*Age (years)*			
25–29^b^	870	43 (4.9)	1
30–34	999	82 (8.2)	1.72 (1.18–2.52)
35–39	1007	71 (7.1)	1.46 (0.99–2.16)
40–44	1150	86 (7.5)	1.56 (1.07–2.27)
45–49	953	61 (6.4)	1.32 (0.88–1.97)
50–54	573	28 (4.9)	0.99 (0.61–1.61)
*P* for trend			0.031
			
*Smoking*			
Ever	3285	238 (7.2)	1.25 (1.01–1.56)
Never^b^	2266	133 (5.9)	1
			
*Marital status*			
Married^b^	5257	343 (6.5)	1
Unmarried^c^	295	28 (9.5)	1.49 (0.99- 2.25)
			
*Lifetime sexual partners*			
1^b^	5378	358 (6.7)	1
⩾2	168	13 (7.7)	1.24 (0.64–2.04)
			
*Husband sex partners*			
1^b^	5270	338 (6.4)	1
2	231	24 (10.4)	1.66 (1.07–2.57)
⩾3	10	2 (20.0)	3.67 (0.77–17.43)
*P* for trend			0.021
			
*Age at first intercourse (years)*			
⩽20	379	38 (10.0)	1.74 (1.14–2.65)
21–27	3939	261 (6.6)	1.10 (0.84–1.46)
⩾28^b^	1226	72 (5.9)	1
*P* for trend			0.027
			
*Pregnant*			
Never	113	12 (10.6)	1.82 (0.98–3.37)
Ever^b^	5438	359 (6.6)	1
			
*Age at first pregnancy (years)*			
⩽24	2613	182 (7.0)	2.09 (1.01–4.32)
25–29	2593	169 (6.5)	2.00 (0.97–4.12)
⩾30^b^	227	8 (3.5)	1
*P* for trend			0.135
			
*Oral contraceptives*			
Never^b^	191	16 (8.4)	1
Ever	5361	355 (6.6)	0.75 (0.39–1.43)
			
*Condom use*			
Never^b^	3525	251 (7.1)	1
Ever	2027	120 (5.9)	0.8 (0.63–0.96)

Abbreviations: OR=odds ratio; CI=confidence interval.

a Adjusted for age.

b Reference category.

c Unmarried includes separated, divorced and widowed.
